# A Histopathological Study of Induced Open Wounds Treated With Urinary Bladder Submucosa Scaffold in Rabbits

**DOI:** 10.1155/vmi/6649280

**Published:** 2026-03-10

**Authors:** Majid A. Alkhilani, Omar Tariq Hammoodi, Ali A. Tala’a, Waleed Al-Nuaimy

**Affiliations:** ^1^ Department of Surgery and Obstetrics, College of Veterinary Medicine, University of Fallujah, Fallujah, Iraq, uofallujah.edu.iq; ^2^ Department of Anatomy and Histology, College of Veterinary Medicine, University of Fallujah, Fallujah, Iraq, uofallujah.edu.iq; ^3^ Department of Electrical Engineering and Electronics, University of Liverpool, Liverpool, UK, liv.ac.uk

**Keywords:** accelerate wound healing, extracellular matrix, natural scaffold, urinary bladder submucosa

## Abstract

Treating various wounds is one of the challenges that researchers are working on to find innovative ways to shorten and accelerate healing. Various materials have been used for this purpose, including those from the submucosal layer of certain animals, such as the calf bladder used in this study. Since extracellular matrix (ECM) contains many components that are important for the healing process, such as collagen, laminin, and hyaluronic acid, it has the potential to accelerate wound healing. The study used twelve adult rabbits. After surgical preparation, a 2 × 2 cm square wound was made on each side of the animal’s body behind the costal arch in the upper abdomen. The wound on the right side was washed with normal saline only as a control group, and the wound on the left side was treated with a dry decellularized ECM. Fresh urinary bladders were collected from slaughtered calves and prepared through mechanical and chemical processes for decellularization. Excess collagenous connective and adipose tissues were removed from the bladder’s external surfaces. The submucosal layer, tunica serosa, and tunica muscular were carefully removed using a knife. The resulting sheet of submucosal bladder was then soaked in phosphate‐buffered saline (PBS) at a pH of 7.4, which contained penicillin (100 IU/mL), streptomycin (100 μg/mL), and amphotericin (100 μg/mL). The decellularized bladder matrix (UBM) was treated with a mixture of 0.1% peracetic acid (PAA) and 4% ethanol, shaking the solution for 2 h at room temperature. The wound was covered with a sterile gauze until the seventh postwounding day (PWD), when the wound was measured, and samples were collected for histopathological examination. Additional samples were collected for histopathological examination at the 14th and 21st PWDs. Visual inspection, wound size measurement, and histopathological examination of both groups revealed that the ECM scaffold had a significant effect on accelerating healing compared with the control group starting from the seventh PWD. The percentage of wound contraction was clearly in favor of the treatment group compared to that of the control group. At the end of the experiment, the epidermal layer and rete ridge were completely thick, with fibroplasia of the dermal layer in the treated group, while in the control group, there was fibrosis in the subcutaneous tissue, and granulation tissue consisting of blood vessels, fibroblasts, and collagen fibers infiltrated with mononuclear cells, but there was no rete bridge. In conclusion, the use of ECM scaffolds plays an important role in accelerating wound healing, making its use advantageous in open wounds.

## 1. Introduction

The skin has many more functions besides basic mechanical protection; it is central to fluid and heat exchange with the environment [1, 2]. A series of interactions occur in response to skin injury, but the main stages are hemostasis, inflammation, formation of new tissue, and remodeling [3]. Migration of polymorphonuclear leukocytes to the wound and macrophages follows to engulf and destroy microbes and necrotic debris and plays an influential role in the healing process of the wound through releasing proteases that decompose damaged extracellular matrix (ECM) and in tissue cytokines [4]. The fibrin matrix and necrotic tissue begin to be replaced by fibroblasts with ECM, which initiates granulation tissue formation, maturation of ECM due to the remodeling phase, including the ECM remodeling into normal skin architecture, and differentiation of some fibroblasts into contractile myofibroblasts, which share in wound contraction [5]. Also, control of infection and inflammation is important to successful wound healing. ECM is composed of many ingredients such as collagen, hyaluronic acid, and laminin. Cells in tissue secrete ECM; these ingredients are arranged in specific tissue patterns, in which collagen is widely used for coating materials and skin tissue engineering. ECM is one of the promising biomaterials for varied applications [6].

Fibroblasts are the dominant cell during the proliferation phase of wound healing. Fibroblasts are critical components of granulation tissue and achieve variant functions, including the manufacture of collagen, elastin, fibronectin, glycosaminoglycan, and proteases. Also, the production of the ECM acts for further critical functions; it promotes the adherence of the cells, significantly manages, and arranges the growth of the cells inside it [7, 8]. Mature fibroblasts are decreased at the last section of this phase and replaced by myofibroblasts [9].

The final phase of the proliferation stage involves enhancing the dense granulation tissue, while the remodeling process has already commenced. This intermediate tissue acts as a substitute for the fibrin and fibronectin‐based temporary wound matrix, which can eventually lead to scar formation through maturation [10, 11]. During the proliferation phase, typically spanning from the 3^rd^ to 10^th^ postwounding day (PWD), the primary goal of the healing process is to protect the surface of the wound, producing granulation tissue and restoring the vascular complex. This is accompanied by the migration of local fibroblasts through the fibrin mesh and the initiation of re‐epithelialization at the wound edges, while neovascularization and angiogenesis begin to occur [12, 13].

Collagen is secreted to the extracellular space in the form of precollagen, which is then transformed into collagen fibrils, which aggregate to form collagen fibers. Elastin is also present in the wound in smaller amounts [14]. The repaired tissue becomes further fibrous through the replacement of Type III collagen by Type I collagen. Then, the collagen fibers are arranged longitudinally in the repaired site; by this means, restoring tensile strength, as well as decreasing the process of angiogenesis, the blood flow descends, and the activity of the acute metabolic rate of the wound slows and eventually stops [15–17].

Many studies on different therapeutic materials have been used to improve and enhance wound healing and maintain internal homeostasis. New techniques, measures, and products were used, including medical dressing and therapy to accelerate the healing of treated wounds [18, 19].

This study aims to evaluate the efficacy of a urinary bladder submucosa scaffold on the healing of experimentally induced open wounds in rabbits.

## 2. Materials and Methods

### 2.1. Ethical Approval

The local committee of animal care at the University of Fallujah was granted ethical approval before starting the study (Number 31 on 21/5/2024).

### 2.2. Materials and Surgical Procedure

**FIGURE 1:**
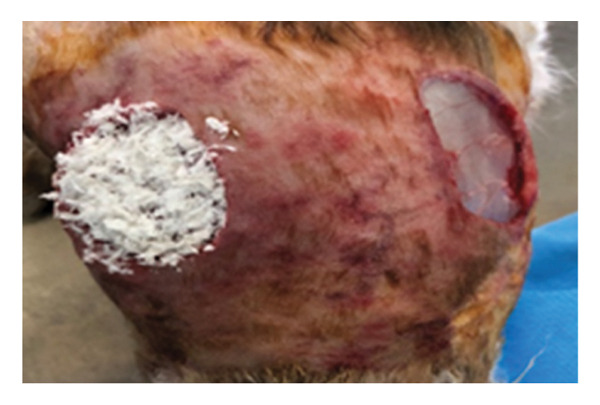
The left induced incision was covered with DSMS, and the right was washed with normal saline.

Twelve adult (9–12 months), local breeds of both sex rabbits (4 females and 8 males), weighing 1.5–2 kg, were included in this research. All rabbits were housed in the animal house of the College of Veterinary Medicine, Al Fallujah University. They were fed green and concentrated food and examined physically to determine that they were healthy. The skin was prepared surgically with an aseptic routine technique, and square full‐thickness skin wounds (2 × 2 cm) were made on all animals in both lateral abdominal regions behind the coastal arch under general anesthesia by using a combination of ketamine (35 mg/kg B.W and xylazine 5 mg/kg B.W, I/M) [20]. The left wounds were treated with decellularized extracellular matrix (DECM) scaffold as a layer covering entire wounds, and the right wounds were not treated and left as control (Figure 1). The wounds were dressed with sterile bandage to prevent any contamination until the specimens were taken for measurement of wound size and for histopathological study on the 7^th^, 14^th^, and 21^st^ PWD; no antibiotics was administered postwounding to all animals in both groups. Specimens of skin were collected on the 7^th^, 14^th^, and 21^st^ PWD, conserved in formaldehyde, and they were prepared in the usual manner for the purpose of histopathological study, sectioned in 6 μ, and stained with H&E [21] in the College of Veterinary Medicine, University of Baghdad.

### 2.3. Preparation of Urinary Bladder Submucosal Scaffold

The decellularized urinary bladder scaffold was prepared following the protocol outlined in references [19, 22, 23]. Fresh urinary bladders were collected from calves at a local abattoir and underwent mechanical and chemical processes for decellularization. Excess collagenous connective tissues and adipose tissue were removed from the bladder’s external surfaces using scissors. The bladder was then bisected to create a rectangular sheet from its opening to the apical region. The submucosal layer, tunica serosa, and tunica muscularis were carefully excised using a knife. The resulting flattened rectangular sheet of the bladder (submucosal layer) was soaked in phosphate‐buffered saline (PBS) at a pH of 7.4, which contained penicillin (100 IU/mL), streptomycin (100 μg/mL), and amphotericin (100 μg/mL). Following this, the decellularized urinary bladder matrix (UBM) was treated with a mixture of 0.1% peracetic acid (PAA) and 4% ethanol, shaking the solution for 2 hours at room temperature to minimize the risk of host rejection. The ECM was then rinsed with PBS and shaken for 15 min at room temperature. It was subsequently rinsed in water twice, again shaken for 15 min at room temperature, and finally rinsed with PBS once more. The resulting decellularized ECM scaffolds were sterilized by immersion in a 0.1% PAA solution titrated to pH 7.0 at room temperature for 5 h. The scaffolds were preserved in sterile PBS containing antibiotics and antifungal agents and then stored at 4°C. The decellularized UBM sheets were allowed to set slightly before being transferred to −20°C for 24 h, followed by storage in a deep freezer at −80°C for 5 days. Subsequently, the scaffolds were freeze‐dried for 4 days at −50°C. After freeze‐drying, the decellularized material was thawed at room temperature and lyophilized before being milled into a fine powder. The powder was sterilized by heating it in an oven at 60°C for 16 h and later stored in a sterile container.

The wound’s length and width are measured by a Vernier caliper, and the percent wound contraction is calculated by the following equation [24]:

(1) percent total wound contraction=wound area of day Noriginal wound area of day 0∗100%.

### 2.4. Statistical Analysis

Two‐way analysis of variance (ANOVA) using the standard least‐squares procedure of JMP Pro 16.00 software (SAS, Institute Inc., Cary, NC, USA) was used to analyze the wound contraction rate. Fisher’s least significant difference (LSD) post hoc test was used to determine the differences between treatment or time at *p* ≤ 0.05.

## 3. Results

All animals tolerated the surgical procedures well, and none of the experimental animals developed outward signs of systemic infection at the surgical site at any time postoperatively.

### 3.1. Macroscopic Findings

**FIGURE 2:**
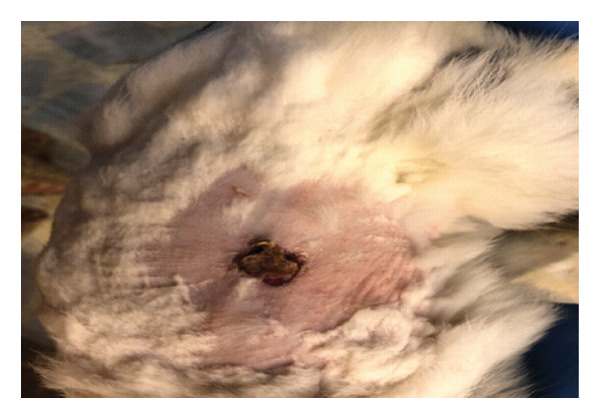
Wound of the control group on the 7^th^ PWD.

**FIGURE 3:**
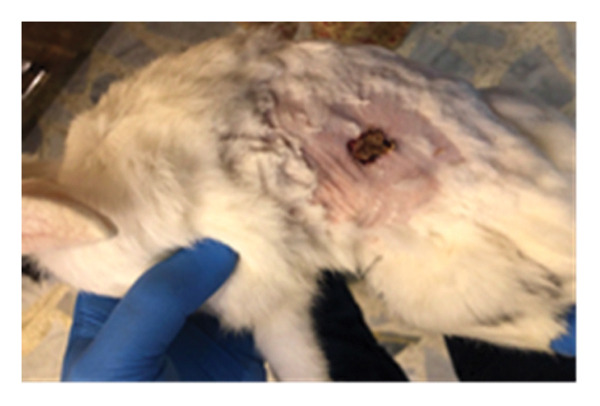
Wound of the treatment group on the 7^th^ PWD.

**FIGURE 4:**
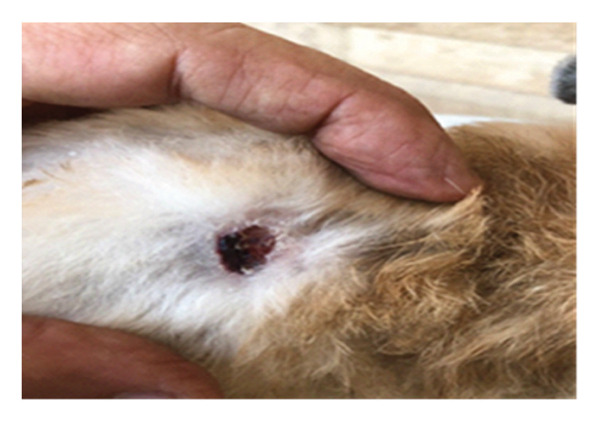
Wound of the control group on the 14^th^ PWD.

**FIGURE 5:**
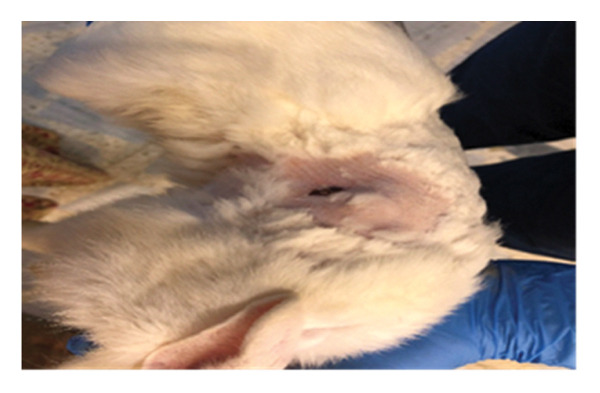
Wound of the treatment group on the 14^th^ PWD.

**FIGURE 6:**
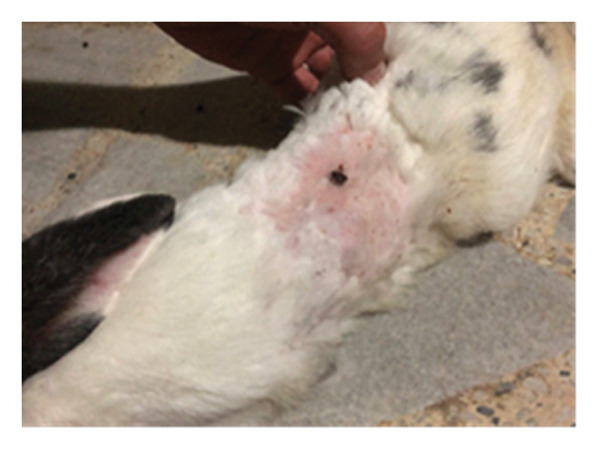
Wound of the control group on the 21^st^ PWD.

**FIGURE 7:**
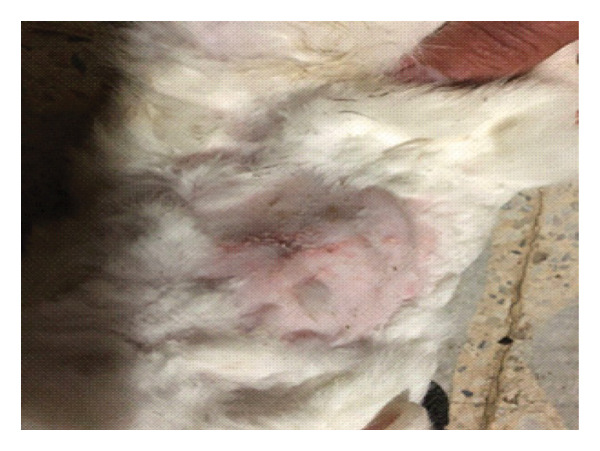
Wound of the treatment group on the 21^st^ PWD.

On the seventh PWD, wound contraction occurred in both groups compared to the first wounding day, with the presence of a scab covering the wound, noting that wound size was significantly reduced in the treated group with DSMS (Figures 2 and 3). The difference in wound size was clearer at the 14^th^ PWD when comparing the two groups (Figures 4 and 5). At the 21^st^ PWD, full epithelialization was observed in the treated group, but in the control group, there was still a scab (Figures 6 and 7), approximately the size of the scab in the treated group at the 14^th^ PWD.

The wound contraction percentage calculated according to the equation of [24] showed that the percentage of wound contraction was clearly in favor of the treatment group compared to that of the control group (Table 1); this was a result of calculating the percentage based on the wound contraction of the wound measurements, as shown in Table 2, which demonstrates the mean value of the wound size in centimeters for all periods in both groups. The mean value for the treated group was 2.4 and 0.37 cm, and the scar formation was at the 7^th^, 14^th^, and 21^st^ PWD, while in the control group, the mean value was 2.8, 1.3, and 0.06 cm at the 7^th^, 14^th^, and 21^st^ PWD, respectively.

**TABLE 1:** Percentage (%) of wound contraction.

Animals	Treatment group				Mean	Control group				Mean
	1^st^ subgroup	2^nd^ subgroup	3^rd^ subgroup	4^th^ subgroup		1^st^ subgroup	2^nd^ subgroup	3^rd^ subgroup	4^th^ subgroup	
Postwound day										
7^th^	30%	25%	30%	20%	26.25	15%	10%	12.5%	10%	11.875
14^th^	75%	70%	72%	65%	70.5	40%	45%	57%	52%	48.5
21^st^	100	100	100	100	100	90%	85%	80%	90%	86.25

**TABLE 2:** Measurement of the wound size by centimeters.

Animals postwound day	Treatment group (cm^2^)					Mean	Control group (cm^2^)				Mean
	Zero day	1st subgroup	2nd subgroup	3rd subgroup	4^th^​ sub group		1^st^ subgroup	2^nd^ subgroup	3^rd^ subgroup	4^th^ subgroup	
7^th^	4	2.24	2.55	2.55	2.4	2.4	2.85	2.5	3	2.88	2.8
14^th^	4	0.35	0.24	0.4	0.49	0.37	1.43	1.68	1.2	0.9	1.3
21^st^	4	scar	scar	scar	scar	—	0.03	0.02	0.08	0.12	0.06

The statistical study showed that the treatment group was superior in wound healing speed throughout the study periods, and wound contraction was significant at *p* < 0.05 (Table 3) and​ (Figure 8).

**TABLE 3:** Effect of urinary bladder submucosa scaffold on wound contraction rate (%).

Time (days)	Treatment		Main effect Time	*p* value (LSD)
	Control	Scaffold		
7	11.9 ± 1.20^f^	26.2 ± 2.39^e^	19.1 ± 2.99^Z^	< 0.001 (4.805)
14	48.5 ± 3.75^d^	70.5 ± 2.10^c^	59.5 ± 4.61^Y^	
21	86.2 ± 2.39^b^	100 ± 0.00^a^	93.1 ± 2.82^X^	
Main effect treatment	48.9 ± 9.26^B^	65.6 ± 9.19^A^		
*p* value (LSD)	< 0.001 (3.924)			
Treatment × time				
*p* value (LSD)	< 0.001 (6.796)			

*Note: n* = 4 per treatment group. Values are means ± SEM, a–f Means followed by different lowercase letters in the same column or row are different from each other in the interaction effect Treatment × time. A‐B Means followed by different uppercase letters in the same column differ in the treatment factor. X‐z Means followed by different uppercase letters in the same column are different in the time factor.

FIGURE 8Effect of factors: (a) time (including the control group and urinary bladder submucosa scaffold group, (b) treatment (including all times), and (c) treatment (including both treatment and times). Values are means ± SEM, *n* = 4.

**Figure:**
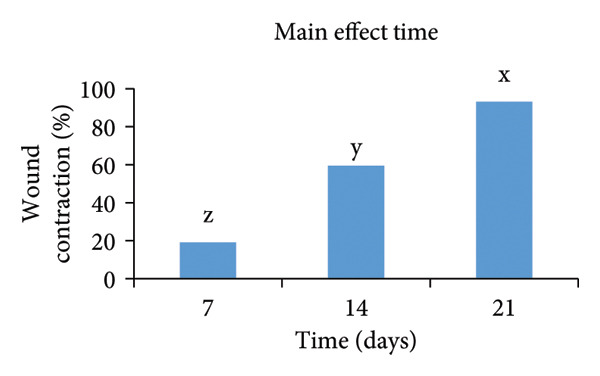
(a)

**Figure:**
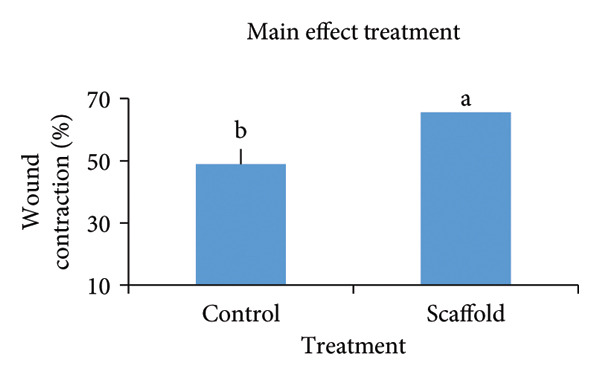
(b)

**Figure:**
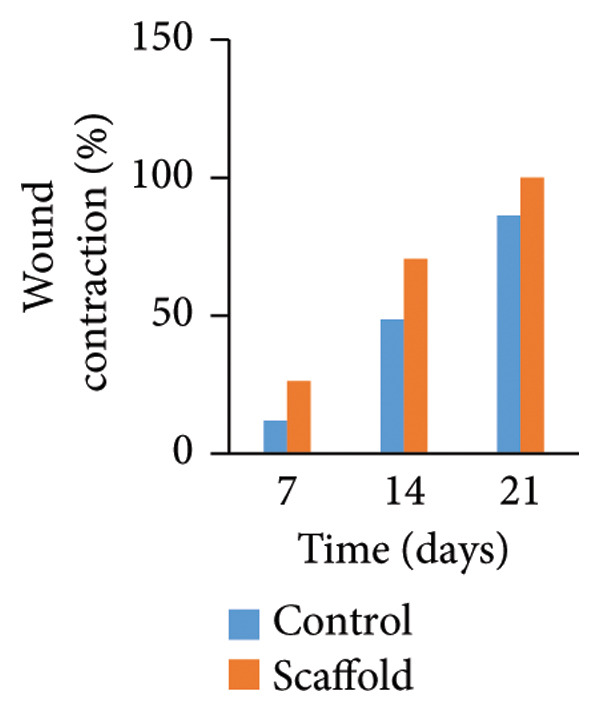
(c)

### 3.2. Histopathological Findings

**FIGURE 9:**
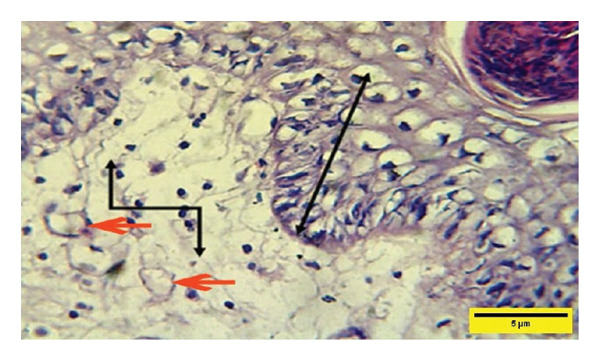
Histopathological section of the treated group on the 7th post‐wounding day shows a vacuolated epidermal layer 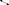 and a high number of inflammatory cells 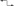 and neovascularization (red arrows) (H&E stain 400 X).

**FIGURE 10:**
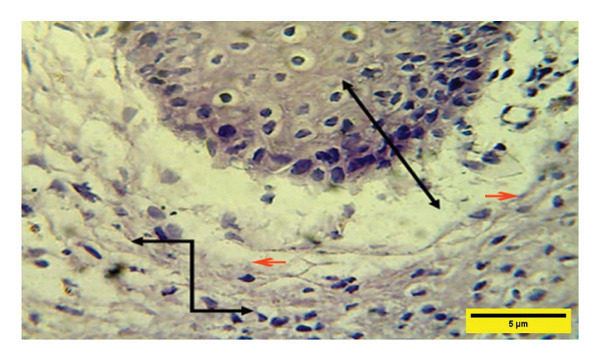
Histopathological section of the treated group on the 7^th^ postwounding​ day shows thick rete ridge 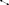 and edema in the subepidermal layer in addition to inflammatory cell aggregation in the dermal layer 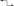 with a high number of fibroblasts (red arrows) (H&E stain 400 ×).

On the 7^th^ PWD, the histopathological picture of the treated group showed a vacuolated thick epidermal layer with the presence of a rete ridge (RR) over the dermal layer, which was infiltrated with mononuclear cells and neovascularization with a high number of fibroblasts (Figures 9 and 10).

**FIGURE 11:**
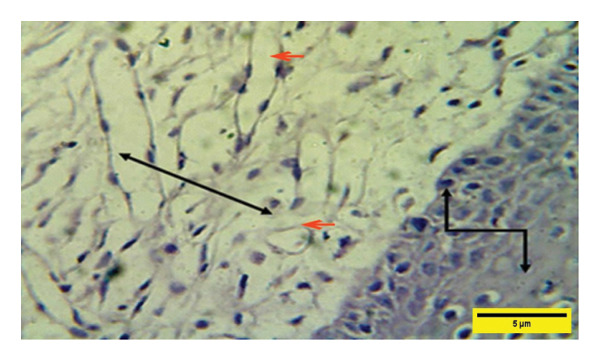
Histopathological section of the control group on the 7^th^ postwounding day shows vacuolation of the epidermal layer 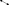 over vascular granulation tissue in the dermis with a high number of inflammatory cells 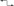 and neovascularization (red arrows) (H&E stain 400 ×).

In the control group, at the same time, the histopathological finding showed a vacuolated epidermal layer, but less than in the treated group on the same day, and vascular granulation tissue in the dermis infiltrated with denser infiltration of inflammatory cells, and no RRs were found with a high concentration of inflammatory cells, and neovascularization (Figure 11).

**FIGURE 12:**
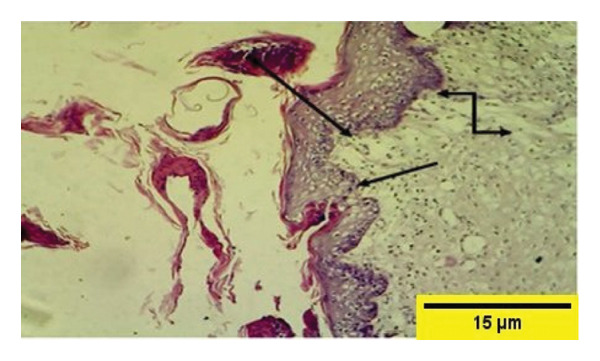
Histopathological section of the treated group on the 14^th^ postwounding day shows a complete thickness epidermal layer 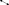 with cellular rete ridge 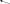 under epidermal layer, and over fibroplasia of dermal layer, collagen fibers appear in some areas dense and regular and fibroblast cells 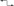 (H&E stain 100 ×).

**FIGURE 13:**
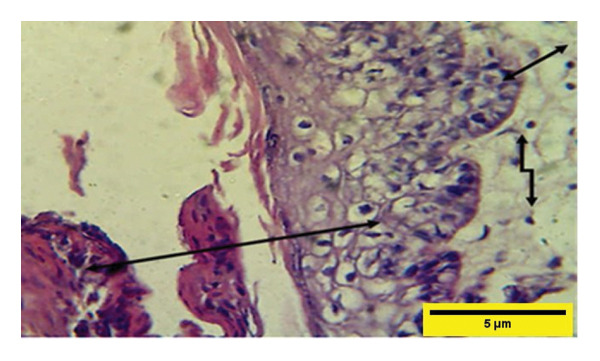
Histopathological section of the treated group on the 14^th^ postwounding day shows a complete thickness epidermal layer 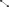 with rete ridge over fibroplasia of the dermal layer infiltrated with a few mononuclear cells 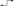 (H&E stain 400 ×).

**FIGURE 14:**
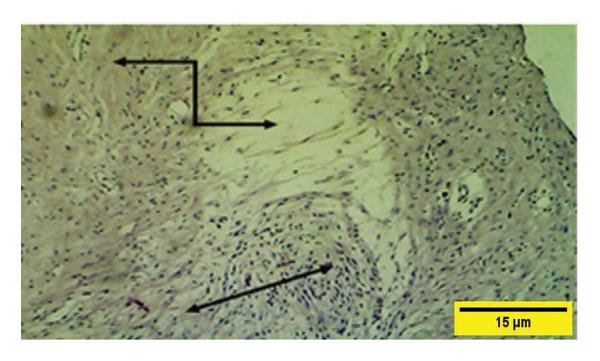
Histopathological section of the control group on the 14^th^ postwounding day shows fibrosis with collagen fibers that were dense, thick, and better arranged around the incision gap in S/C tissue 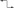 infiltrated by mononuclear cells 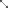 (H&E stain 100 ×).

**FIGURE 15:**
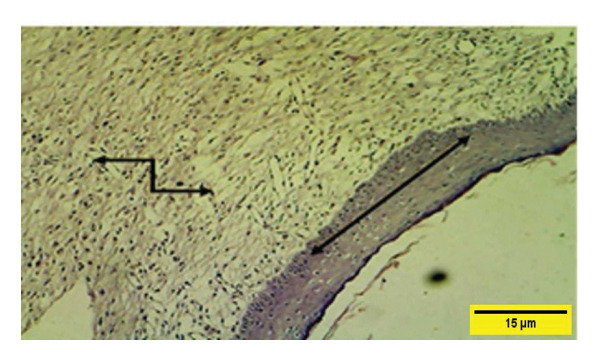
Histopathological section of the control group on the 14^th^ postwounding day shows collagen fibers developed and well arranged in the epidermal layer 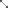 over vascular granulation tissue consisting of blood vessels, fibroblasts, and fewer inflammatory cells 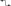 (H&E stain 100 ×).

**FIGURE 16:**
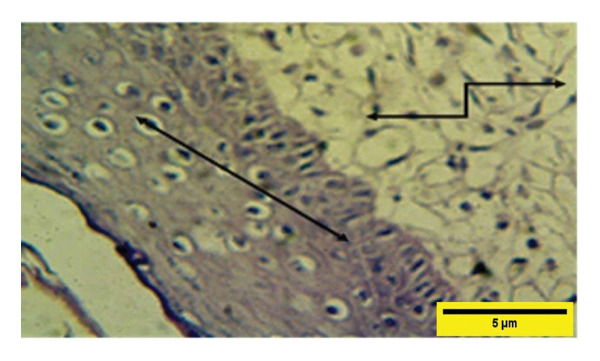
Histopathological section of the control group on the 14^th^ postwounding day shows vacuolation of the epidermal layer 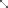 over vascular granulation tissue in the dermis infiltrated by inflammatory cells 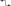 (H&E stain 400 ×).

On the 14^th^ PWD in the treated group, there was the complete thickness of the epidermal layer, and RR, with fibroplasia of the dermal layer, and collagen fibers appeared in some areas dense and regular, and fibroblast cells (Figures 12 and 13). While in the control group on the 14^th^ PWD, there was fibrosis in the subcutaneous tissue, and granulation tissue consisting of blood vessels, fibroblasts, and collagen fibers appeared irregular and not arranged in parallel, infiltrated with mononuclear cells, but there was no RR (Figures 14, 15 and 16).

**FIGURE 17:**
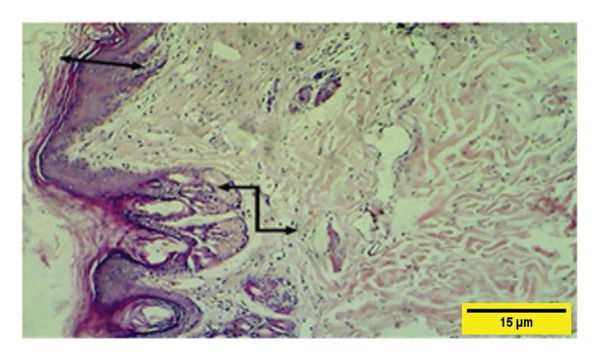
Histopathological section of the treated group on the 21^st^ postwounding day shows a complete thickness epidermal layer with rete ridge 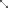, keratinized layer, and collagen fibers that were dense, thick, and better arranged at this stage 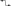 (H&E stain 100 ×).

On the 21^st^ PWD in the treated group, the epidermal layer thickness was complete with a RR and a keratinized layer with sebaceous in the dermal layer; the collagen fibers appear more regular and are arranged in parallel (Figure 17).

**FIGURE 18:**
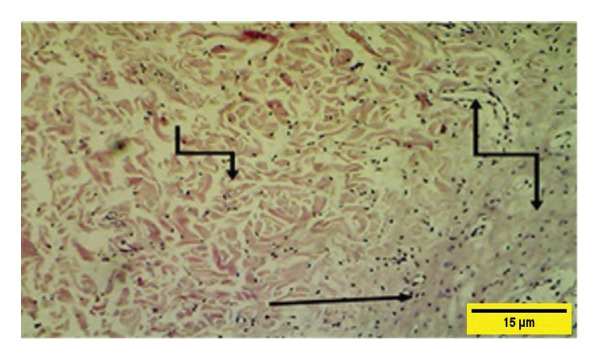
Histopathological section of the control group on the 21^st^ postwounding day shows that collagen fibers were dense and well developed 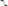, and scattered inflammatory cells 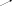 are attached to the muscle layer 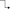 (H&E stain 100 ×).

In the control group on the 21^st^ PWD, vascular granulation tissue appeared to consist of blood vessels, fibroblasts, and collagen fibers infiltrated with mononuclear cells and collagen fiber that till now more of them appear irregular and less dense (Figure 18). The collagen fibers were present in both groups at this time but they were more regular and mature in the treatment group.

## 4. Discussion

The wound size began to contract gradually through the days of experiments as an indicator of the healing process development, but it was better in the treated group as a result of the ECM used. This agrees with many researchers, including [9], who noticed that the wound contraction in the treated group with methionine was faster and earlier than in the control group, which began on the 7^th^ PWD. Likewise, the researchers [25] saw that the size of the wound decreased significantly on the 14^th^ PWD to more than 50% compared to the size of the wound on zero PWD.

Skin tissue regeneration is a significant clinical challenge because of the various cell types and the complex structure of the skin. Traditional methods, such as cell transplantation and growth factor treatments, frequently fail due to the adverse conditions present at the wound site [26].

The epidermal layer in the treatment group appeared thicker when compared to that in the control group from the 7^th^ PWD, and its thickness was complete on the 14th PWD in the treatment group compared to that in the control group. The fibroblasts appeared on day 7 as part of the natural wound healing process, producing collagen fibers, which was clearly present on the 14th day PWD in both groups; it was more regular with a wavey shape in the treatment group, but it was not so in the control group, in which the presence of granulation tissue persisted with a dense presence of inflammatory cells on the 14^th^ PWD. This agrees with Al‐Mutheffer et al. [27], who noticed that the epithelial layer reached full thickness on the 14^th^ PWD in the treatment group, compared to that in the control group, which showed granulation tissue and blood vessels at the same time. Weller et al. [28] showed that fibroblasts experienced phenotypic changes and transformed into myofibroblasts, establishing strong adhesions with the surrounding granulation tissue and supplying the force necessary for wound contraction.

Certain areas of the epidermis extend into the dermis, creating a wavy formation known as the RR. The dermis, as the most resilient fibrous layer of the skin, is primarily composed of collagen fibers, fibroblasts, blood vessels, nerves, and immune cells [28]. RRs are important microstructures at the dermis–epidermis junction in the skin, crucial for enhancing skin mechanics and homeostasis. In tissue engineering and regenerative medicine, while artificial skin grafts have advanced, the restoration of RRs is often overlooked, possibly affecting the effectiveness of skin healing and regeneration [29]. In this study, the RRs were seen in the treatment group from the 7^th^ PWD but were not present in the control group on the 7^th^ and 14^th^ PWD or even the 21^st^ PWD, which explains the healing process that agrees with [30, 31] as the morphology of RRs is a key characteristic of both intact and regenerated human skin, and the depth and structure of RRs serve as indicators of the effectiveness of wound healing [32].

The clear, dense collagen fibers on the 14th PWD and mature and regular on the 21st PWD in the treatment group in this study were indicated to advance in wound healing as [33] who found that the active components within different substances were used to enhance the wound healing process by improving the durability of collagen fibrils, strengthening collagen fibers through increased circulation, and preventing cell damage. The authors [34] explain that collagen often accumulates during the final phase of wound healing, and the collagen fibers were visible on either side of the wound; then, they spread to the center, and ultimately, they were organized uniformly throughout the whole dermis. Furthermore, the regeneration of skin, collagen, and blood vessels increased in the treatment group, which is consistent with [35].

The scaffolds ought to permit the transport of the supplements required for the cell connection, multiplication, and differentiation; incitement of cell–biomaterial connection, development, and relocation; and a controlled debasement rate with no harmfulness or aggravation hazard to the cells [36]; therefore, the results of this study showed a clear advantage for wound healing in the group treated with scaffold when compared to that in the control group through the advanced healing stages since the 7^th^ PWD, and this indicates a clear effect of ECM in wound healing in a shorter period. This ECM attracts the inflammatory cells like neutrophils, lymphocytes, and macrophages, which infiltrate the defect. The neutrophils were the main cells in an acute inflammatory stage, which play an important role in phagocytosis. Also, the infiltrated macrophages play an important role in delivering growth factors and cytokines to facilitate the conversion to the proliferative stage, which agrees with Al‐Mutheffer et al. [27] who proved that the use of some treatments like DL‐methionine, which was used in their study, accelerates the maturation and remodeling process, which should actually begin when the connective tissue matures and the granulation tissue turns into a scar, and [25] noticed that after 14 days of infection, the treated wounds with blue led light have a higher degree of epithelialization.

Our study agreed with Alkhilani et al. [19] who found that at day 21^st^ PWD, healing was evident in the treated groups, with good epithelialization associated with newly produced mature collagen fibers and angiogenesis, while the control group still had poor epithelialization with inflammatory cells and mild angiogenesis. The occurrence of advanced healing in these groups is due to the presence of the ECM scaffold, which provides a fundamental part of repair and healing.

## 5. Conclusion

The healing time in the treatment group was earlier and clearer on the 7^th^ PWD, which indicates a good effect of the ECM scaffold on accelerating healing. The use of an ECM scaffold, which is a natural substance, in the treatment of open wounds was effective in the rate of wound healing and contraction.

## Funding

No funding was received for this manuscript.

## Conflicts of Interest

The authors declare no conflicts of interest.

## Data Availability

The data that support the findings of this study are available from the corresponding author upon reasonable request.
